# The Association Between Early Administration of Noninvasive Ventilation and Short-Term Outcome for Acute Heart Failure

**DOI:** 10.7759/cureus.18909

**Published:** 2021-10-19

**Authors:** Taichi Nakazawa, Hiraku Funakoshi, Chinami Sakurai, Koki Iwata, Satsuki Yamazaki, Yosuke Homma, Tetsuya Inoue

**Affiliations:** 1 Department of Emergency and Critical Care Medicine, Tokyo Bay Urayasu Ichikawa Medical Center, Urayasu, JPN

**Keywords:** respiratory failure, oxygenation, noninvasive positive pressure ventilation, dyspnea, acute decompensated heart failure

## Abstract

Background

Acute decompensated heart failure (ADHF) is a life-threatening disease that requires emergent intervention. Although noninvasive positive pressure ventilation (NPPV) is crucial for treating ADHF, the earliest time point for administering NPPV remains unknown. In this study, we hypothesized that early NPPV administration for patients with acute heart failure in the emergency department (ED) would lead to a better outcome.

Methodology

This is a single-center retrospective cohort study at an ED of a community hospital in Japan. The data were collected from consecutive patients who were administered NPPV for ADHF in the ED from April 2016 to September 2018. The primary exposure was the timing of NPPV administration (within 30 minutes versus over 30 minutes after arrival). The primary outcome was 30-day mortality.

Results

A total of 115 patients were included in this study. Overall, the median age was 78 (interquartile range [IQR] = 70-84 years), and 63 (54.9%) patients were male. The median time from the arrival at the ED to NPPV administration for the patients was 14 minutes (IQR = 8-30 minutes). Overall, 72% (83/115) of the patients were categorized as early administration group (<30 minutes). The total 30-day mortality was 7.0% (8/115), and the total tracheal intubation rate was 11% (13/115). Early NPPV administration for patients with ADHF was associated with lower 30-day mortality (3.6% vs. 16%; p = 0.04) and shorter length of oxygenation (four days vs. seven days; p < 0.01). Multivariate logistic regression test showed that 30-day mortality was significantly lower in the early treatment group (adjusted odds ratio = 0.19; 95% confidential interval = 0.04-0.90).

Conclusions

Although further investigation is needed, early NPPV administration for patients with ADHF in the ED was associated with lower 30-day mortality.

## Introduction

Acute decompensated heart failure (ADHF) is a life-threatening disease and a common medical emergency that accounts for up to one million hospital admissions for acute conditions each year in the United States [1]. In addition, because in-hospital mortality associated with ADHF is still high [2], establishing effective treatment is an important issue worldwide.

Both the American Heart Association (AHA) and the European Society of Cardiology (ESC) guidelines recommend the use of noninvasive positive pressure ventilation (NPPV) for patients with ADHF [3,4]. Moreover, a systematic review has shown that NPPV for patients with ADHF can reduce mortality and tracheal intubation rate [5]. In addition, NPPV plays a key role in not only improving oxygenation but also reducing the work of breathing and increasing cardiac output [6-8].

In addition, guidelines have also emphasized the importance of immediate diagnosis and treatment of patients with ADHF. A recent study has shown that early intravenous administration of vasoactive drugs and diuretics may reduce mortality [9,10].^ ^However, whether earlier administration of NPPV upon arrival of patients at the emergency department will affect the mortality of patients with ADHF remains unclear. Therefore, in this study, we aimed to investigate the association between early NPPV administration and the outcome of patients with ADHF.

## Materials and methods

Study design and setting

This is a single-center retrospective cohort study conducted at the emergency department (ED) of a community hospital, a 344­­-bed urban acute care community hospital in eastern Tokyo, which is a regional trauma center and is designated as a 24-hour stroke/cardiovascular center with the capability of percutaneous coronary artery intervention.

This study was approved by the ethics committee of the hospital and was conducted according to the ethical guidelines of the Declaration of Helsinki. The patients’ information was anonymized and deidentified before the analysis, and the informed consent of the patients was waived.

Selection of participants

Patients who received NPPV due to ADHF in the ED between April 1, 2016, and September 31, 2018, were included in the study. We diagnosed ADHF according to the Framingham criteria. Patients under the age of 18 years, non-ADHF patients treated with NPPV, or patients with missing records of NPPV administration time were excluded.

Study protocol

ADHF was diagnosed by the board of certified emergency physicians based on patients’ history, physical examination (Framingham criteria), laboratory testing including B-type natriuretic peptide (BNP) or N-terminal pro-B-type natriuretic peptide (NT-proBNP), ultrasound sonography, and chest X-ray [11]. All staff treated patients with heart failure according to the recommendations in the guidelines. All patients were treated at the discretion of the treating physicians. The individual protocols regarding the policy and procedures for ADHF management were maintained by each patient. After admission to the ward, patients were treated by a team of general internal medicine doctors with support from the department of cardiology, as needed. The treatment plan remained consistent during the hospitalization. The collected variables include age, sex, respiratory rate, systolic blood pressure, blood gas analysis (pH), ejection fraction observed in ED, Killip classification, and mode of arrival (ambulance or others).

The primary exposure was the door to NPPV time which was defined as the time from hospital arrival to NPPV administration. The early administration group was defined as the door to NPPV time of <30 minutes, and the delayed administration group was defined as the door to NPPV time of ≥30 minutes [12,13]. NPPV was delivered via a face mask.

The primary outcome was 30-day mortality. Secondary outcomes were the occurrence of tracheal intubation and length of oxygenation. The duration of oxygenation was defined as days until the oxygen administration was no longer needed. Patients who temporarily discontinued oxygen administration but restarted it within 24 hours were considered to be on continuous oxygen administration.

Statistical analysis

The characteristics and outcomes of patients were evaluated using Fisher’s exact test for categorical variables and the Mann-Whitney U test for continuous variables. Comparison of the 30-day mortality rate and tracheal intubation in each group was performed using a multivariable logistic regression test. Pertinent confounders were determined based on the clinical expertise of the investigators: Killip classification, which indicates the severity of heart failure; and pH in blood gas, which is an indicator of ventilation failure as well as poor oxygenation. Competing risk analysis was used to compare the length of oxygenation during admission of both groups. The cumulative incidences of the oxygenation-free period were calculated and compared using the competing risk-adjusted model (the Gray method) and the modified Kaplan-Meier method, respectively [14].

The statistical analysis was performed using EZR (Saitama Medical Center, Jichi Medical University, Saitama, Japan), which is a graphical user interface for R 3.3.4 (R Foundation for Statistical Computing, Vienna, Austria) that adds frequently used statistical functions in biostatistics [15]. Two-sided P-values of <0.05 were considered statistically significant.

## Results

During this study, 66,277 patients visited the ED. Of these, 1,756 patients were treated as ADHF patients, and 121 patients with ADHF were administered NPPV in the ED. We excluded six patients because of missing data, leaving 115 patients in the analysis. The baseline characteristics of the included patients are summarized in Table 1. Overall, the median age was 78 years (interquartile range [IQR] = 70-84 years), and 54% of the patients were male. A total of 84 patients were classified as Killip classification three or four. The median door to NPPV time was 14 minutes (IQR = 8-30 minutes). Based on this result, 72% (83/115) of the patients were categorized as the early administration group. The total 30-day mortality was 7.0% (8/115), and the total tracheal intubation rate was 11% (13/115). The median duration of oxygenation was three days (IQR = 3-8 days). Patients in the early administration group had significantly higher systolic blood pressure, more severe acidemia, higher Killip classification, and were more frequently transported by ambulance. The cause of ADHF, treatment, mode of NPPV, and the other variables were not significantly different.

**Table 1:** Characteristics of patient with heart failure characteristics according to the door to NPPV time groups. IQR: interquartile range; NPPV: noninvasive positive pressure ventilation; EF: ejection fraction; CPAP: continuous positive airway pressure; BiPAP: bilevel positive airway pressure Data are expressed as n (%) unless otherwise indicated. Total percentages may not be 100 due to rounding or duplication.

Patient characteristics	Total (n = 115)	Early administration group (n = 83)	Delayed administration group (n = 32)	P-value	
Age, median (IQR), year	78 (70–84)	78 (70–82)	81 (71–85)	0.13	
Male sex	62 (54%)	47 (57%)	15 (47%)	0.4	
Respiration rate >25 breaths per minute	79 (69%)	59 (71%)	20 (63%)	0.38	
Systolic blood pressure >140 mmHg	93 (81%)	72 (87%)	21 (66%)	0.02	
					pH <7.2	22 (19%)	20 (24%)	2 (6.2%)	0.03	
EF, median (IQR), %	45 (34–57)	46 (35–56)	43 (31–59)	0.48	
Killip classification 3 or 4	84 (73%)	68 (82%)	16 (50%)	0.01	
Transport by ambulance	96 (83%)	74 (89%)	22 (68%)	0.01	
Cause of heart failure					
Acute coronary disease	10 (9%)	8 (10%)	2 (6%)	0.72	
Acute valve failure	2 (1.7%)	2 (2.4%)	0 (0%)	1	
Anemia	3 (2.6%)	2 (2.4%)	1 (3.1%)	1	
Arrhythmia	14 (12%)	8 (9.6%)	6 (19%)	0.21	
Congestion	57 (51%)	42 (51%)	17 (53%)	0.84	
Hypertensive emergency	63 (55%)	49 (59%)	16 (50%)	0.41	
Treatment					
Furosemide	85 (74%)	59 (71%)	26 (81%)	0.35	
Nitroglycerine	87 (76%)	68 (82%)	19 (59%)	0.02	
Nicaldipine	3 (2.6%)	2 (2.4%)	1 (3.1%)	1	
Intervention or operation	12 (10%)	9 (11%)	3 (9.4%)	1	
Hemodialysis	16 (14%)	14 (17%)	2 (6.2%)	0.23	
Mode of NPPV					
CPAP	39 (34%)	24 (29%)	15 (47%)	0.08	
BiPAP	76 (66%)	59 (71%)	17 (53%)	0.08	
Door to NPPV time, median (IQR), minutes	14 (8–30)	10 (5.0–10)	53 (39–92)	<0.01	
30-day mortality	8 (7.0%)	3 (3.6%)	5 (16%)	0.04	
Tracheal intubation	13 (11%)	9 (11%)	4 (13%)	0.08	
Duration of oxygenation, median (IQR), day	3 (3–8)	4 (3–6)	7 (5–11)	<0.01	
Duration of ventilation, median (IQR), day	1.0 (1.0–2.0)	1.0 (1.0–1.5)	1.0 (1.0–3.0)	0.06	

**Figure 1:**
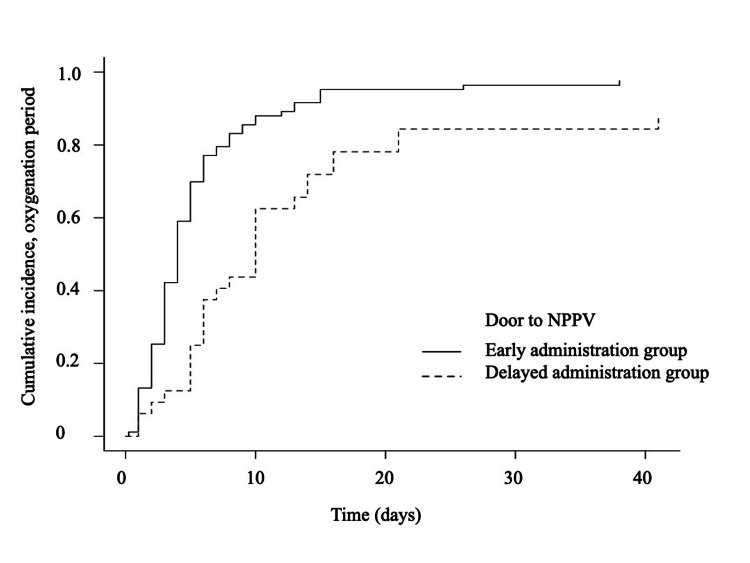
The modified Kaplan-Meier curve for the duration of oxygenation in the early administration of NPPV (<30 minutes) group and the delayed administration of NPPV (≥30 minutes) group. The cumulative incidence rate of oxygenation-free period among early administration of NPPV (<30 minutes) or delayed administration of NPPV (≥30 minutes). NPPV: noninvasive positive pressure ventilation

The median door to NPPV time was 10 minutes (IQR = 5.0-10 minutes) in the early administration group and 53 minutes (IQR = 39-92 minutes) in the delayed administration group. Univariate logistic regression test showed that 30-day mortality was significantly lower in the early treatment group (odds ratio [OR] = 0.20; 95% confidential interval [CI] = 0.045-0.90). This association remained significant after adjusting for predefined potential confounders (adjusted OR = 0.19; 95% CI = 0.039-0.90) (Table 2). In contrast, the occurrence of tracheal intubation was not significantly different in both groups (OR = 1.17; 95% CI = 0.34-4.12). The competing risk analysis indicated that the length of oxygenation was significantly shorter in the early administration group (four days, IQR = 3-5 days) than in the delayed administration group (10 days, IQR = 6-14 days). The cumulative incidence of oxygenation-free period adjusted for the competing risk-of-mortality plot was significantly different between the two groups (modified log-rank, P < 0.01) (Figure 1).

**Table 2:** Associations between the time to administration of NPPV and 30-day mortality among patients with ADHF. ADHF: acute decompensated heart failure; CI: confidence intervals; NPPV: noninvasive positive pressure ventilation; OR: odds ratio

Variables	Unadjusted OR (95% CI)	P value	Adjusted OR (95% CI)	P-value
Door to NPPV time ≥30 minutes vs. <30 minutes	0.20 (0.045–0.90)	0.04	0.19 (0.039–0.90)	0.04
Covariates				
Killip classification	-	-	1.45(0.36–5.83)	0.60
pH <7.2	-	-	0.89(0.09–8.52)	0.91

## Discussion

In this retrospective study, early NPPV administration for patients with ADHF was associated with lower 30-day mortality and shorter length of oxygenation, regardless of the severity of patients in the early administration group. On the other hand, the rate of intubation did not significantly differ.

Recent studies have shown the impact of early NPPV administration on patients with ADHF. The ADHERE study suggested that early nitride use for patients with ADHF was significantly associated with a shorter duration of hospital stay and lower risk for prolonged hospital stay (P < 0.01) [9]. In addition, early intravenous vasodilatory drug use can improve in-hospital mortality. A recent prospective multicenter study suggested that early administration of intravenous diuretics such as furosemide within one hour after admission can reduce in-hospital mortality [10]. According to these studies, early initiation of treatment is important for a better prognosis of patients with ADHF. These results corroborated our findings that early administration of NPPV can contribute to better patient outcomes.

Patients with ADHF frequently present with dyspnea, hypoxemia, and anxiety caused by acute cardiogenic pulmonary edema following increased back pressure on the pulmonary circulation. In addition, the sympathetic nervous system activated by severe symptoms leads to tachycardia, hypertension, peripheral vasoconstriction, and diaphoresis, which worsen acute cardiogenic pulmonary edema [16]. In addition to improving oxygenation, NPPV can reduce the work of breathing and increase the cardiac output by maintaining positive airway pressure during the respiratory cycle that prevents alveolar collapse at the end-expiration [7,16]. Furthermore, positive end-expiratory pressure increases intrathoracic pressure and decreases preload by reducing venous return to the heart. Increased intrathoracic pressure reduces left ventricular intramural pressure and afterload. The activated sympathetic nervous system is suppressed with improved oxygenation and breathing, which subsequently reduces afterload [16]. As described above, NPPV has a positive effect not only on oxygenation but also on many exacerbating conditions such as increased preload and afterload. Thus, early NPPV administration can affect patients’ prognoses. Our study could provide more efficient care for patients with ADHF by supporting the concept of time-dependent treatment. Once the importance of early administration is clarified, the next step is to determine which factors are involved in the delayed introduction, which will lead to further improvement in the quality of medical care.

There are several limitations in the current study. First, this was a single-center study, which might affect its generalizability. Second, due to the limited number of patients, a multivariable analysis could not be conducted. Therefore, potential confounders (age, sex, and vital signs) were not adjusted. The code status (e.g., Do Not Attempt Resuscitation and Do Not Intubate) could influence the patients’ outcome [17]. Patients in the early NPPV group had high blood pressure, worse acidemia, and a higher proportion of Killip classification grade 3 or 4. In contrast, patients in the delayed NPPV group had lower blood pressure with relatively compensated acidosis, possibly due to underlying chronic heart failure. These two groups could be different in terms of the pathophysiology of heart failure. Third, there are potentially unmeasured confounders due to the retrospective nature of the study, such as the main cause of ADHF (e.g., acute coronary syndrome) and chronic kidney disease with hemodialysis. Fourth, the data were collected from medical records, which may lead to reporting bias. Fifth, we did not include patients who were administered NPPV after admission to the general ward. The patients administered NPPV after admission could have relatively mild symptoms. Excluding those patients could overestimate the severity of patients in the delayed administration group, leading to selection bias. However, our aim in this study was to evaluate the association between early NPPV administration for patients with ADHF at the ED and mortality rates during the acute phase. Sixth, we did not have data on NPPV mode and settings, such as driving pressure or fraction of inspired oxygen. While some studies have shown that continuous positive airway pressure (CPAP) leads to a more favorable outcome, both CPAP and bilevel ventilation can improve patient outcomes. This might affect the outcome of our study; however, doctors in our hospital changed the mode to synchronize patients to NPPV.

## Conclusions

Our results show that early NPPV administration for ADHF is associated with 30-day mortality and the duration of oxygenation. Although the study has several limitations, NPPV can be a time-dependent treatment option for patients with ADHF. For an emergency physician, this result supports the concept of early NPPV administration. As this study was a single-center retrospective analysis with a small sample size, further prospective studies are needed.
